# The Role of Skin Barrier in the Pathogenesis of Food Allergy

**DOI:** 10.3390/children2030382

**Published:** 2015-09-02

**Authors:** Neema Izadi, Minnelly Luu, Peck Y. Ong, Jonathan S. Tam

**Affiliations:** 1 Department of Pediatrics, Children’s Hospital Los Angeles, Los Angeles, CA 90027, USA; E-Mail: nizadi@chla.usc.edu; 2 Department of Dermatology, Keck School of Medicine, University of Southern California; Los Angeles, CA 90033, USA; E-mail: mluu@chla.usc.edu; 3 Division of Clinical Immunology and Allergy, Department of Pediatrics, Children’s Hospital Los Angeles, Keck School of Medicine, University of Southern California; Los Angeles, CA 90027, USA; E-Mail: pyong@chla.usc.edu

**Keywords:** food allergy, skin barrier, allergic sensitization, filaggrin

## Abstract

Food allergy is a serious public health problem with an increasing prevalence. Current management is limited to food avoidance and emergency treatment. Research into the pathogenesis of food allergy has helped to shape our understanding of how patients become sensitized to an allergen. Classically, food sensitization was thought to occur through the gastrointestinal tract, but alternative routes of sensitization are being explored, specifically through the skin. Damaged skin barrier may play a crucial role in the development of food sensitization. Better understanding of how patients initially become sensitized may help lead to the development of a safe and effective treatment for food allergies or better prevention strategies.

## 1. Introduction

Food allergy represents a serious public health problem, with recent studies showing an increased prevalence in both North America and the United Kingdom. Although an exact figure is difficult to obtain, current estimates show that food allergy affects approximately 6%–8% of young children and 3%–4% of adults in the United States [1]. The increase in prevalence has led to an unsurprising increase in anaphylaxis and hospitalizations [2,3]. Unfortunately, current therapeutic options are limited to avoidance of the culprit allergen and injectable epinephrine in case of accidental exposure.

Most individuals with food allergy are otherwise in good health; however, the unpredictable and episodic nature of acute and potentially life-threatening food-allergic reactions may lead to significant distress for affected children and their parents or caregivers [4,5]. Furthermore, food allergy results in significant medical costs for the US health care system, especially in families with a food-allergic child [6]. Considering the rising prevalence, negative effects on quality of life, and financial burdens of food allergy, there is significant interest in understanding the mechanisms behind food allergy to help guide and develop therapies.

Recent research has focused on modifying risk of developing food allergy through improved understanding of factors, such as timing and route of exposure that lead to allergic sensitization *versus* tolerance. Sensitization refers to the development of an immune state in which antigen exposure leads to an immune response with the potential for hypersensitivity with re-exposure. By contrast, tolerance refers to the immune state in which no significant immune response is mounted upon re-exposure [7]. Food allergy must begin with an initial sensitization event to the food protein; however, the route and timing by which this sensitization occurs currently remain unclear. Classically, food sensitization was thought to occur primarily through the gastrointestinal tract [8], but alternative routes of sensitization are currently being explored, specifically through the skin. Here we review the mechanisms of allergic sensitization to foods and their implications for the future directions of food allergy prevention and treatment.

## 2. Oral Tolerance and Allergic Sensitization through the Gut

Despite the extent of foreign protein exposure, relatively few patients have food allergies due to the development of oral tolerance. Oral tolerance is the state of unresponsiveness to everyday ingestion of harmless antigens and is produced by the inherent mechanical process of digestion as well as specific mechanisms of immune suppression [9]. Disturbances at various steps along the pathway of oral tolerance result in food sensitization and food allergy.

Oral tolerance begins with the basic process of digestion and absorption, which prevents most food antigens from presentation to the immune system. Food proteins that enter the gut are digested by proteases and absorbed as a mixture of free amino acids and peptides. Proteins that escape digestion predominantly pass through the gut without incident due to the intact mucosal barrier of the gastrointestinal tract and partial degradation by gastric acid [10,11,12]. Infants secrete less stomach acid and have less pancreatic enzyme output compared to adults [13]. Combined with a somewhat increased intestinal permeability [14,15], this increases the chances of intact allergen crossing the epithelial border and the risk for sensitization and allergy in infants. Interestingly, clinical observations and murine models indicate that treatment with antacid medications may also increase the risk of sensitization to ingested foods [16].

For proteins that bypass the protective mechanisms of digestion, oral tolerance may still occur due to mechanisms of unresponsiveness mediated by the gastrointestinal immune system. Active sampling of intestinal antigen helps to regulate immune responses and to ensure intestinal homeostasis. Gut sampling of food proteins can occur through multiple mechanisms including intestinal epithelial cells (IECs), microfold cells (M cells), or directly by macrophages and dendritic cells (DCs). IECs and M cells both release factors important for the development of oral tolerance. IECs can package and export food antigens to be sampled by professional antigen presenting cells (APCs) in the *lamina propria* [17,18], or act as nonprofessional APCs and present antigen themselves directly to T cells [19,20]. IEC involvement results in tolerance due to unique factors that dampen the immune response and promote gut homeostasis [21].

The relationship between allergic response and other types of immune regulation is dependent on T cell control. Specific T helper (Th) subsets dictate cytokine production and the regulation of these responses. Classically, Th2 inflammatory responses typify the allergic response involving immunoglobulin (Ig)E production and eosinophilic infiltration as a result of the actions of interleukin (IL)-4, IL-5, and IL-13 [22]. Another subset of T cells characterized by high levels of CD25 expression (IL-2 receptor (R) α chain) have been identified as regulatory T cell (Tregs), because they were found to suppress the function of other T cells when present in the same site [23]. These CD4+CD25+ Tregs are pivotal in oral tolerance and active immune regulation [24]. Interactions between different aspects of the mucosal immune system with Tregs make up a large portion of the current understanding for mechanisms in oral tolerance.

For example, IECs have also been shown to aid in the generation of tolerogenic dendritic cells and subsequently Tregs [25]. Moreover, classic APCs like macrophages and DCs in the gut lead to immune suppression mainly through specific Treg differentiation and production of anti-inflammatory cytokines like IL-10 [26,27]. Macrophages and DCs can also directly sample antigen by sending out protrusions between epithelial cells [28,29], and then migrate to mesenteric lymph nodes (MLNs) in a C-C chemokine receptor (CCR)7 dependent manner [26,30]. CD11c^+^CD103^+^CX3CR1^−^ DCs decrease sensitization through Flt3 (FMS-like tyrosine kinase-3) ligand mediated expansion [31], and release transforming growth factor (TGF)-β, and retinoic acid leading to expansion of Foxp3+ (forkhead box P3+) T regulatory cells [24,31,32,33,34,35,36].

M Cells line the apices of Peyer’s patches and specialize in the uptake of particulate antigens, delivering them to APCs. They lack a brush border and have a deeply invaginated surface for easy uptake of antigen to be presented to APCs in the membrane pocket or in local gut-associated lymphoid tissue (GALT) for TGF-β mediated IgA switching and differentiation of Tregs [37,38,39,40,41]. IgA has been shown to play an adjunct role to prevent sensitization through binding of antigen to prevent systemic absorption [24,42]. Mice deficient in the ability to secrete IgA and IgM were much more sensitive to IgG-mediated anaphylaxis [43]; however, mice could still be orally tolerized despite low levels of fecal IgA production suggesting IgA is helpful, but not necessary to oral tolerance [44].

Immunological as well as genetic evidence support Tregs role in oral tolerance. Children with a history of milk allergy who went on to develop tolerance had higher frequency of CD4+CD25+ Tregs compared to those who maintained clinically active allergy [45]. Further, children who recently developed natural tolerance to their egg or peanut allergy showed higher number of Tregs compared to allergic or non-allergic control patients [46], further suggesting an important role for regulatory T cell subsets in the acquisition of natural tolerance. Mutations in the *FoxP3* gene result in absent or abnormal Tregs and lead to the syndrome IPEX (immune dysregulation, polyendocrinopathy, X-linked) [47,48]. These patients with IPEX develop food allergies and eczema demonstrating a failure of tolerance. Murine studies show that depletion of Tregs alone leads to loss of oral tolerance [26,32]. Moreover, in mice Tregs are sufficient to diminish the allergy response [49], likely through secretion of the inhibitory molecules such as glucocorticoid-induced TNFR (Tumor necrosis factor receptor) family related gene (GITR), CTLA4 (cytotoxic T-lymphocyte-associated protein 4) [50], TGF-β, and IL-10 [51] and the downregulation of pro-effector T cell cytokines such as IL-2 [52,53,54,55]

## 3. Allergic Sensitization through the Skin

Several clinical observations support the existence of sensitization routes other than the gut. For example, allergic reactions to foods have been noted to occur without prior ingestion exposure [56,57], and in general, oral exposure to foods are limited in infancy and lead to tolerance [58,59]. While the potential role for exposure in utero or through breast milk has been proposed, there is some conflicting evidence that suggests that these exposures may more likely induce tolerance rather than sensitization [60,61,62]. Taken together, this evidence suggests that another route other than perinatal and oral may be important to the development of food allergies.

Recently, the skin has garnered significant interest as a potential important route for allergic sensitization. Epidemiologic data suggests that sensitization to peanut protein can occur in children via exposure of inflamed skin to peanut oils [63]. In a study by Lack *et al.*, the risk of allergic sensitization to peanut in children with atopic dermatitis (AD) was not associated with maternal peanut ingestion during pregnancy or lactation; however development of peanut allergy was significantly increased in those infants using skin creams containing peanut oil [63].

Furthermore, a study comparing children with peanut allergy, children with egg allergy (high risk control group), and children without allergy found a dose-dependent relationship between household peanut consumption and the development of peanut allergy [64]. There was no difference in peanut consumption between the three groups of children suggesting that increased household consumption led to increased allergy to peanut via another route, possibly exposure to peanut dust [64]. Household peanut dust is known to directly correlate with household consumption, and in fact multivariate regression analysis has shown household peanut dust to correlate most closely with peanut protein levels in the infant’s environment (crib, play area, sheets, *etc.*) [65,66]. Moreover, a dose dependent increase in sensitization with peanut dust levels was observed in patients with atopic dermatitis and fillagrin loss-of-function mutations [67,68]. This further suggests a transdermal route of sensitization given the impaired skin barrier in these populations.

Although peanut dust exposure can occur via the airway, current evidence supports the skin as a more likely route for sensitization. Higher levels of skin homing receptors on peanut-reactive T cells suggest that the initial peanut sensitization event occurs in the skin. Studies in patients with peanut allergy have shown T cells with high expression of skin homing receptor CLA (cutaneous leucocyte-associated antigen receptor) in the serum [69]. Additionally peanut component Ara h1 reactive T cells in the serum of peanut allergic individuals expressed high levels of CCR4, another skin homing receptor [70].

Like the mucosal immune system and gastrointestinal tract, the skin barrier plays a critical role in host defense against microbial invasion and allergen exposure. The skin epidermis is a highly complex, dynamic, self-renewing barrier against the external environment. The *stratum corneum* (SC), the outermost layer of the epidermis, is a sturdy barrier comprised of crossed-linked matrix containing lipids and proteins produced by keratinocytes, which minimize both water loss and protect from microbial or allergen penetration. Tight junctions (TJs) act as an intercellular sealing apparatus responsible for the compartmentalization of extracellular environments [71]. The TJ barrier acts as a divider between two liquid compartments. However, TJs are not just physical barriers. In fact, they exhibit ion and size selectivity that allows for dynamic regulation of substances crossing between compartments.

Filaggrin (FLG) is an important protein of the skin and serves several crucial functions to maintain the integrity of the skin barrier. Filaggrin is a highly insoluble 37-kD protein that derives from a more abundant 400 kD proprotein, profilaggrin [72]. Profilaggrin is the main component of keratohyalin granules in the *stratum granulosum* (SG) of the epidermis. During terminal differentiation of the keratinocyte, proteases process profilaggrin into monomers [73] that function to align and assemble keratin intermediate filaments into macrofibrils, thus providing integrity and strength to the SC [74,75,76].

Filaggrin is also involved in the crucial natural moisture and acidic environment of the skin. Filaggrin monomers are broken down into amino acids and other derivatives that readily attract moisture to form natural moisturizing factor (NMF) [77,78], hygroscopic compounds important for SC hydration. FLG and its derivatives, particularly urocanic acid, play important roles in maintaining an acidic skin pH. This acidic pH environment modulates serine proteases [79], which effect lipid assembly and SC organization both *in vitro* [78,80,81,82] and in murine models [83,84,85]. The acidic environment maintained by filaggrin has also been shown to provide a protective state against bacterial colonization, specifically *Staphylococcus aureus.* Key proteins important for *S. aureus* colonization, such as iron-regulated surface determinant A, are downregulated by the acidic environment provided by filaggrin in intact skin [86]. In addition, the skin barrier defect in AD leads to increased binding protein secretion and more efficient colonization by *S. aureus* [86]. Not surprisingly, over 90% of patients with AD are colonized with *S. aureus* compared to only 5% in healthy individuals [87]. Preventing *S. aureus* growth is important as increased colonization can lead to increased sensitization through bacterial superantigens and an overall heightened immune state from *S. aureus* proteins [87,88].

The importance of filaggrin deficiency in allergic sensitization has been shown in several murine studies using the filaggrin loss of function mutation (*FLG*) flaky tail (ft/ft) mouse. In a key study of these flaky tail mice, ovalbumin (OVA) was applied to the skin to elicit inflammatory infiltrates and enhanced allergen priming [89]. While the normal mice did not develop any specific IgE response, the flaky tail mice showed elevated OVA-specific IgE without any additional adjuvant or abrasion of the skin. However, transmembrane protein79 (*Tmeme79/matt*) gene, rather than *FLG*, was later proven to be responsible for the spontaneous development of dermatitis—in the flaky tail mouse model [90,91]. *Tmem79/matt* gene encodes for lamella granular proteins, which are responsible for processing filaggrin, lipids, proteases and antimicrobial peptides.

*FLG* has been associated with increased food allergy and several diseases with known barrier defects such as *ichthyosis vulgaris* and AD [80]. Depending on the study, *FLG* has been found in high percentages of up to 56% in these conditions [92]. In food allergy, the impaired skin barrier resulting from defects in filaggrin expression has been hypothesized as a gateway for food allergens and as a way to avoid the oral tolerance pathways of the gut mucosa [93]. This may also explain the connection between early severe atopic dermatitis and food allergy. In fact, *FLG* loss of function has been directly associated with human food allergy. An analysis of 71 peanut allergic patients from Europe as compared to 1000 non-allergic patients showed a strong and statistically significant connection between the occurrence of the allergy and *FLG*. This risk was further replicated in analysis of the results from 390 Canadian patients [94]. A large 18-year birth cohort showed a strong association between fillagrin loss-of-function mutation and food allergy only when patients also developed eczema [95]. This suggests that increased food allergy in fillagrin deficiency is mainly due to the barrier dysfunction of eczema, rather than a direct interaction between fillagrin and the immune system. In another cohort, a similar indirect effect of fillagrin loss-of-function was demonstrated for all ages except in the first year of life [96]. This further suggests the primary role of fillagrin in barrier integrity given that the barrier defects of eczema can precede the clinical diagnosis in infancy [97].

Disruptions of barrier function other than filaggrin deficiency are generally required for epicutanous sensitization. In most mouse models of epicutanous sensitization, the SC needs to be mechanically impaired by tape-stripping, patch dressing, or treatment with adjuvant [98,99]. Mouse models of skin sensitization with adjuvant or barrier disruption have shown potent Th-2 response through high IL-4 and IgE in response to peanut sensitization [100], and a long-lasting dose dependent IgE response to hazelnut sensitization [101,102]. Furthermore, a murine study comparing all forms of sensitization with adjuvant (intragastric, cutaneous, intranasal, and sublingual) found maximal sensitization to the cutaneous route of exposure [103]. The one notable exception to the requirement of barrier disruption appears to be for peanut. In mice, peanut skin exposure alone without any skin stripping promotes a Th2-dependent sensitization to peanut allergens [104]. This special adjuvant activity to peanuts, not only shows the unique nature of peanut allergy, but also underscores the skin as a key site of sensitization.

Skin sensitization, although not as widely studied as the gut, most likely involves thymic stromal lymphopoetin (TSLP). Many studies in the skin suggest TSLP plays a significant role in sensitization. TSLP is an IL-7 like type I cytokine predominantly expressed by epithelial cells [21]. *In vitro* evidence suggests increased expression in the lesional skin of AD patients [105], and TSLP has been shown to potentiate DCs to preferentially differentiate naïve T cells to Th2 cells [106,107]. Similarly, skin specific overexpression of TSLP in a transgenic mouse model demonstrated a dramatic Th2 inflammatory response including increased Th2 CD4+ T cells and elevated IgE [108]. TSLP has also been shown to effect DCs migration to the skin. DCs acquired skin specific migration signals through a TSLP-induced CCR7 and C-X-C chemokine receptor (CXCR)4-dependant pathway leading to contact hypersensitivity in mice [109,110], further demonstrating TSLP role in skin sensitization.

Another important downstream mediator of skin sensitization is OX40L and IL-33. As in the gastrointestinal mucosa, TSLP-activated DCs express OX40L [107], a member of the TNF superfamily implicated in the communication between B cells and T cells [111]. OX40L has been shown to be necessary for the Th2 inflammatory response in mouse models of oral sensitization [112], and recent studies suggest OX40L is also important in skin sensitization mainly through IL-33. In peanut sensitization, OX40L is upregulated in an IL-33 dependent manner [113], and skin-selective IL-33 alone leads to significantly increased Th2-mediated AD-like inflammation in mice [114]. IL-33 has been shown to exacerbate anaphylaxis through mast cell degranulation [115,116], and blocking IL-33 prevents anaphylaxis in cutaneously sensitized mice [117]. Thus, IL-33 and OX40L may represent potential targets for cutaneous sensitization to food antigens in future therapies.

Basophils have recently been implicated as important mediator of skin sensitization. Recent studies have demonstrated that TSLP can drive basophil-dependent Th2 cytokine response [118,119], IgE increase, and accumulation of mast cells in the intestine promoting food allergy [120]. Furthermore, mice epicutaneously sensitized to OVA were protected from oral challenge and did not produce OVA-specific IgE in basophil-depleted or TSLP-receptor-deficient mice, further highlighting the role of basophils and TSLP in skin sensitization [117]. Basophils likely mediate their Th2 effect either as non-professional APCs by presenting antigen themselves [118] or cooperatively with DCs to mediate the downstream production of IL-4 [121,122], leading to increased anaphylaxis, marked mastocytosis, and less tolerance in murine studies [123,124].

The phenomenon of skin sensitization leading to an oral allergic response has been recently coined the “dual-allergen-exposure” hypothesis [125]. This hypothesis further proposes that exposure to food antigens through the skin is more likely to lead to allergy compared to early oral consumption, which is more likely to lead to tolerance. The previously discussed tendency of the gut mucosa to elicit tolerance compared to the more sensitizing mechanisms of the skin makes a strong case for this hypothesis. Furthermore, what ultimately determines food allergy is likely a combination of both skin and gut exposure to a particular food antigen with a higher tendency toward sensitization if the first exposure is through the skin. Given the difficulty of eliminating food allergy once it is established, primary prevention based on this hypothesis becomes important.

Clinical evidence demonstrating the validity of the dual-allergen hypothesis led the American Academy of Pediatrics in 2008 to shift its position on food exposure in infancy to favor early oral exposure to allergic foods, thus reversing prior recommendations of waiting past infancy for children at risk for atopic disease [61]. For many years, exclusive breastfeeding and careful elimination diets were considered the best methods for prevention of food allergy. However, introducing previously avoided foods such as cow’s milk, egg, fish, wheat, sesame and peanut has been recently studied in a large cohort of exclusively breastfed infants with no deleterious effects or disruptions in breastfeeding [126]. This lack of evidence and a continued rise in food allergy prevalence suggested that these preventative methods were ineffective. More recent evidence argues that early oral exposure to establish oral tolerance might be a more prudent approach [125,127]. Evidence supporting the role of early exposure in decreasing food allergy has been well demonstrated with peanut allergy [128]. In fact, a recent randomized study done by the Learning Early about Peanut Allergy group demonstrated a dramatic difference in peanut allergy prevalence of 17.2% in the avoidance group compared to only 3.2% in the consumption group [59].

## 4. Future Directions for the Treatment of Food Allergy

The current standard of food avoidance for prevention of food allergy is difficult given frequent accidental exposures and the potential deleterious effects on quality of life. However, new desensitization therapies are currently on the rise. As stated previously, the present standard of care is strict dietary avoidance as there is currently no approved treatment or disease-modifying therapy for the routine management of patients with food allergies. Food avoidance strategies are not only difficult but they also pose the risk of malnutrition [129], poor growth [130], accidental exposure, and decreased quality of life in children [131,132,133]. The most promising therapy in development for the effective treatment of food allergy is desensitization through controlled antigen exposure involving oral or sublingual immunotherapy (OIT and SLIT) [134,135]. Both SLIT (peanut, milk, hazelnut, fruit, peach), and OIT (peanut, milk, egg) have been shown to be efficacious in reducing food allergy through several studies [135,136]. SLIT is considered safer as smaller quantities are used in the mouth and many important allergen epitopes are digested in the stomach, while OIT can use higher doses making it more efficacious but also more prone to adverse reactions. This comparison between SLIT and OIT has been shown both retrospectively with peanut allergy [137] and in a recent open-label randomized human study of milk tolerance, where OIT showed significantly more desensitization but at the cost of more systemic side effects [138]. Given the higher rate of desensitization OIT is generally considered the preferred route of immunotherapy [135].

The exact mechanism of desensitization from immunotherapy is not completely understood but changes in the levels of immunoglobulins and FoxP3 expression (Tregs) have been implicated. Several potential immune mechanisms have been proposed including: decreasing levels of specific IgE, increased specific IgG, decreased reactivity of basophils and mast cells, and increased Tregs [139,140]. In addition, a recent study using patients with peanut allergy found increased specific Tregs activity and hypomethylation of FoxP3 sites in immune tolerant individuals when compared to non-tolerant; however, no significant difference was found in peanut specific IgE or basophil activation [141]. Thus, OIT induced epigenetic modifications, mainly hypomethylation, may lead to increased FoxP3 activity and be pro-tolerogenic.

Since skin is a likely site of sensitization, it seemed natural that it could be used as a point of treatment. Epicutaneous immunotherapy (EPIT) utilizes the placement of a patch of soluble allergen directly on intact skin. This approach is not only noninvasive but has been shown to have very minimal side effects [142]. The efficacy of EPIT was first established in mouse models. Mice sensitized to peanut who were given EPIT showed significantly reduced Th2 cytokines, reduced IgE response, increased specific IgG, and increased FoxP3 mRNA after oral challenge when compared to placebo mice [143]. Further evidence of EPIT efficacy was found with pollen, house dust mite, and OVA exposure; however, when compared to traditional subcutaneous immunotherapy (SCIT) there was no significant difference in decreasing the IgE/IgG ratio [144]. Similar equivalence between EPIT and SCIT was also observed in mice again sensitized to peanut antigen [145].

Mouse models have also helped us understand the possible mechanism of desensitization through intact skin. One study of mice using OVA EPIT demonstrated that prolonged application leads to antigen capture by DCs in the SC with transportation to draining lymph nodes [146]. Furthermore, repeated exposure to OVA down-modulated specific and systemic responses with an associated induction of Tregs [146]. In a recent study, still in progress, EPIT significantly decreased the Th2 immune response and increased FoxP3^+^ Tregs [147]. Two other very recent studies, also works in progress, have shown increased gut homing receptors CXCR3, CCR6, CCR8 in response to EPIT but not SLIT or OIT [148], and increased CCR6 and CCR9 with antigen-specific latency-associated peptide^+^, FoxP3^+^ that was absent in OIT-treated mice [149]. This increased gut homing mediated by EPIT may represent a desensitization advantage over other forms of therapy given the gut mucosa’s predilection toward tolerance.

Preliminary evidence of EPIT in human trials has shown some promise. The largest human study to date is a single-centered, placebo-controlled, double-blind trial of grass pollen sensitization where 37 adult patients were found to have decreased nasal provocation tests compared to placebo by the 2^nd^ year of EPIT and significantly decreased skin prick response with only 10% having mild local reactions requiring antihistamines [150]. More recently, a bicenter, double-blind placebo-controlled (DBPC) study of children with oral challenge positive milk allergy showed good tolerability but showed little change in food challenge after three months, although similar oral immunotherapy studies have shown that benefits become more apparent after more than one year [142]. However, given the acceptable safety profile, a comprehensive plan to develop EPIT for peanut allergy by DBV Technologies has made great strides. After completing a DBPC phase I safety trial with only mild adverse reactions, a subsequent DBPC phase IIa trial showed promising results with up to 67% desensitization response for 54 children with severe peanut allergy at 18 months and four subjects even tolerating doses of up to 3.3 to 8 peanuts [151]. A follow up DBPC phase IIb dose-finding trial running in 22 centers in the United States and Europe reached its primary endpoint using Viaskin brand Peanut at a 250 microgram dose where 53.6% of children responded to treatment compared to 19.4% in the placebo group at 12 months with increased peanut-specific IgG by over 19 times, and IgE increasing at first but then decreasing toward initial levels [152]. This study demonstrates the potential of EPIT and hopefully increased focus on EPIT for other allergies will lead to more well-established convenient routes of desensitization in the future. Lastly, recent studies have shown that skin barrier therapy may prevent *atopic dermatitis* [153,154]. Whether such therapy may lead to the prevention of food allergy in atopic dermatitis patients remains to be an area of interest for future investigations.

## 5. Summary

Food allergy is a serious public health concern with the most established therapies limited to food avoidance and emergency treatment. To develop better therapies, it is important to understand how the route of exposure may determine sensitization *vs.* tolerance. The damaged skin barrier may play a crucial role in the development of food sensitization. As we learn more about the process of sensitization through the skin, it seems like a natural extension to use it as a mode of treatment. Both murine and human EPIT studies have shown promise, but more research is needed to establish both safety and efficacy. But perhaps more importantly, as more is learned about the origins of sensitization, there might one day be a way of preventing the allergy altogether.
